# Experience of rescue therapy with [^177^Lu]Lu-rhPSMA-10.1 in patients with primary or acquired resistance to [^177^Lu]Lu-PSMA-I&T

**DOI:** 10.1007/s00259-024-06959-5

**Published:** 2024-11-04

**Authors:** Alexander Gäble, Alexander Dierks, Andreas Rinscheid, Marianne Patt, Georgine Wienand, Christian H. Pfob, Malte Kircher, Kazuhito Fukushima, Ana Antić Nikolić, Johanna S. Enke, Tilman Janzen, Julie Steinestel, Hildegard Kempter, Martin Trepel, Dorothea Weckermann, Constantin Lapa, Ralph A. Bundschuh

**Affiliations:** 1 Nuclear Medicine, Faculty of Medicine, University of Augsburg, Augsburg, Germany; 2 Bavarian Cancer Research Center (BZKF), Augsburg, Germany; 3 Medical Physics and Radiation Protection, University Hospital Augsburg, Augsburg, Germany; 4 Radiology, Kobe International Collaboration Clinic, Kobe, Japan; 5 Urology, Faculty of Medicine, University of Augsburg, Augsburg, Germany; 6 Internal Medicine, Oncology, Faculty of Medicine, University of Augsburg, Augsburg, Germany

**Keywords:** Prostate cancer, Radioligand therapy, Prostate-specific membrane antigen, Therapeutic response, Radiohybrid ligands

## Abstract

**Purpose:**

Radioligand therapy is an increasingly important option for the treatment of metastatic castrate-resistant prostate cancer (mCRPC). Radiohybrid ligands targeting prostate-specific membrane antigen (PSMA) are a novel group of theranostic radioligand therapy agents for which higher tumour absorbed radiation doses have been demonstrated compared to established PSMA ligands. Here, we report data from ten patients who were treated within a compassionate use program with the radiohybrid PSMA-ligand [^177^Lu]Lu-rhPSMA-10.1 after experiencing disease progression under treatment with [^177^Lu]Lu-PSMA-I&T.

**Methods:**

Ten patients with advanced PSMA-positive prostate cancer who showed progression under treatment with [^177^Lu]Lu-PSMA-I&T received up to three cycles of rescue therapy with [^177^Lu]Lu-rhPSMA-10.1 (7.4–8.1 GBq per cycle). Efficacy (PSA response according to PCWG3 and RECIP) and overall survival were evaluated. Adverse events were recorded from first application.

**Results:**

Despite progression with [^177^Lu]Lu-PSMA-I&T, after the first cycle of [^177^Lu]Lu-rhPSMA-10.1 rescue therapy, five patients (50%) showed a decrease in serum PSA level. In imaging, three of the ten patients (30%) showed a partial radiologic response. Four of the five patients with a decrease of serum PSA under [^177^Lu]Lu-rhPSMA-10.1 had initially responded to treatment with [^177^Lu]Lu-PSMA-I&T but had become resistant. However, the remaining patient had shown continuous disease progression during [^177^Lu]Lu-PSMA-I&T therapy but showed an immediate response to [^177^Lu]Lu-rhPSMA-10.1. The additional treatment with [^177^Lu]Lu-rhPSMA-10.1 was generally well tolerated by all patients.

**Conclusions:**

Patients showing tumour progression while receiving [^177^Lu]Lu-PSMA-I&T radioligand therapy may benefit from rescue therapy with the novel radiohybrid PSMA ligand, [^177^Lu]Lu-rhPSMA-10.1. Higher tumour absorbed radiation doses with [^177^Lu]Lu-rhPSMA-10.1 may overcome primary and acquired radiation resistance.

**Graphical Abstract:**

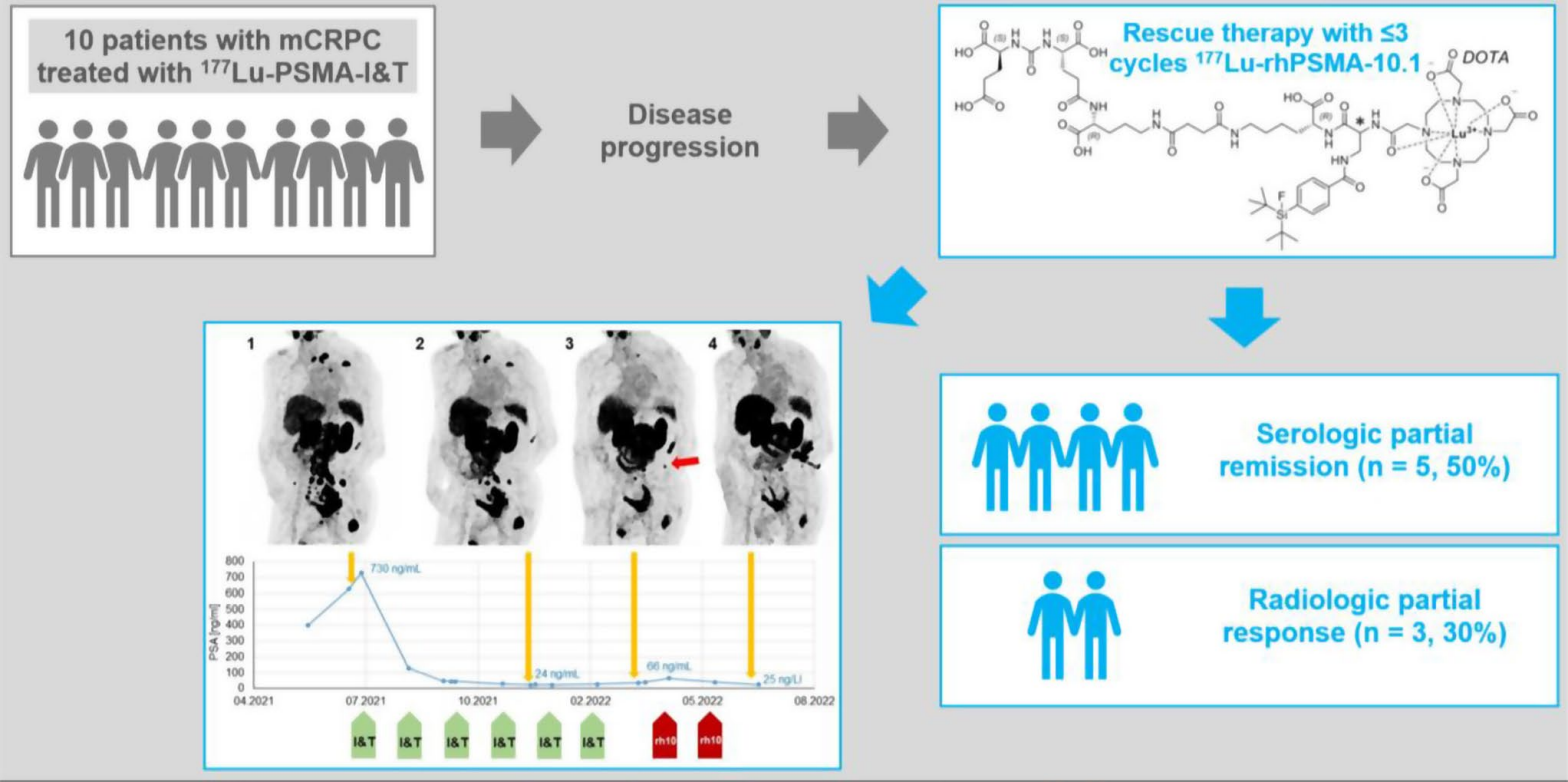

## Introduction

Lutethium-177(^177^Lu)-labelled prostate-specific membrane antigen (PSMA)-targeted radioligand therapies are increasingly being used for patients with metastatic castrate-resistant prostate cancer (mCRPC) who have experienced disease progression on conventional treatments such as novel androgen axis drugs or chemotherapy. Clinical data show promising outcomes with both [^177^Lu]Lu-PSMA-I&T and [^177^Lu]Lu-PSMA-617, with data from the Phase 3 VISION trial showing a survival benefit of [^177^Lu]Lu-PSMA-617 compared with standard of care treatment in patients with mCRPC [1, 2].

[^177^Lu]Lu-rhPSMA-10.1 is an investigational radiopharmaceutical that has been developed using a novel radiohybrid (rh) technology platform that enables engineering of PSMA-targeted ligands that can be labelled with fluorine-18 (^18^F) for diagnostic imaging, or with alpha- or beta-emitting radiometals for radioligand therapy [3]. Following preclinical data that show [^177^Lu]Lu-rhPSMA-10.1 to be a promising candidate for radioligand therapy [4, 5], the safety and anti-tumour efficacy of [^177^Lu]Lu-rhPSMA-10.1 in patients with mCRPC is currently being investigated in a Phase I/II trial (NCT05413850, first registration date: May 20th 2022).

We recently reported the first clinical data with [^177^Lu]Lu-rhPSMA-10.1 from an intra-patient comparison with [^177^Lu]Lu-PSMA-I&T in patients with mCRPC [6]. Our pre-therapeutic dosimetry data demonstrate that [^177^Lu]Lu-rhPSMA-10.1 delivers an up to 8-fold greater radiation dose to the tumour compared with [^177^Lu]Lu-PSMA-I&T [6], which may be of clinical relevance when considering data obtained with [^177^Lu]Lu-PSMA-617 that suggest a better response to treatment is achieved when a greater radiation dose is delivered to the tumour [7, 8]. We further showed [^177^Lu]Lu-rhPSMA-10.1 to have a more favourable tumour-to-kidney therapeutic index than [^177^Lu]Lu-PSMA-I&T [6]. Given that the kidneys are a significant organ-at-risk in patients undergoing radioligand therapy [9], this finding suggests potential for a further clinical benefit through reduction of the radiation exposure to the kidney while still achieving an effective tumour dose. Data from a second report from this cohort show that following 4–6 cycles of [^177^Lu]Lu-rhPSMA-10.1 radioligand therapy, the patients showed a PSA response of between 35% and 100%, with one of the four patients showing a sustained complete response sustained at 32 months (time of writing) [10].

To further delineate the potential clinical utility of [^177^Lu]Lu-rhPSMA-10.1, here, we report data from a series of ten patients with advanced mCRPC who underwent rescue therapy with [^177^Lu]Lu-rhPSMA-10.1 within a compassionate use program after experiencing disease progression while undergoing [^177^Lu]Lu-PSMA-I&T radioligand therapy.

## Materials and methods

### Radiopharmaceutical preparation and approval

This study was conducted in accordance with the Helsinki Declaration and with national regulations. The local institutional review board (review board of the Ludwig-Maximilians-Universität München, Munich, Germany) approved this analysis (permit number 22-1011). [^177^Lu]Lu-rhPSMA-10.1 was prepared in compliance with the German Medicinal Products Act, AMG§ 13 2b, and after informing the responsible regulatory body. All patients gave written informed consent to the imaging and therapeutic procedures.

### Patients and lesions

Ten consecutive patients with advanced and heavily pre-treated mCRPC were included in this analysis after switching to treatment with [^177^Lu]Lu-rhPSMA-10.1 under a compassionate use program following recommendation by the local multidisciplinary tumour team. Eligible patients had castration resistant PSMA-positive metastatic prostate cancer, defined by the presence of at least one PSMA-positive metastatic lesion on [^68^Ga]Ga-PSMA-I&T PET/CT as well as the absence of metastases, with increased uptake in [^18^F]F-fluorodeoxyglucose (FDG) uptake without corresponding PSMA-uptake according to the TheraP trial criteria [11].

The patients had previously undergone multiple therapies including surgery, radiation therapy, androgen deprivation, novel androgen axis drugs, and chemotherapy, as well as radioligand therapy using [^177^Lu]Lu-PSMA-I&T. All patients had most recently shown disease progression (defined according to the Prostate Cancer Clinical Trials Working Group 3– PCWG3 criteria [12]) while undergoing treatment with [^177^Lu]Lu-PSMA-I&T.

### [177Lu]Lu-rhPSMA-10.1 therapy and response assessment

Following an interval of 7 to 12 weeks from the last [^177^Lu]Lu-PSMA-I&T cycle, and of 3 to 8 weeks (median 5.2 weeks) after the last [^68^Ga]Ga-PSMA-I&T PET/CT the patients received 1–3 cycles of [^177^Lu]Lu-rhPSMA-10.1 with a mean dose of (7.44 ± 0.06) GBq per cycle) with an interval of 6 weeks between cycles.

Prostate-specific antigen (PSA) values were monitored as a measure of efficacy using PCWG3 criteria [12], at a minimum of every 6 weeks. Following the second [^177^Lu]Lu-rhPSMA-10.1 cycle, a [^68^Ga]Ga-PSMA-I&T PET/CT was performed for radiographic response assessment using the Response Evaluation Criteria in PSMA PET/CT (RECIP) [13].

### Safety

All adverse events and treatment-related adverse events were recorded and graded according to Common Terminology Criteria for Adverse Events (CTCAE) v5.0 [14] from the first cycle of treatment up to 14 months post-treatment.

### Statistics

The reported data are mainly descriptive. All continuous data are reported as mean, standard deviation, and range.

## Results

### Patients

A series of ten consecutive patients were included in the analysis. Their clinical characteristics are presented in Table 1.

**Table 1:** Clinical characteristics

Patient	1	2	3	4	5	6	7	8	9	10
**ECOG Performance Score**	2	1	1	1	1	1	1	1	1	1
**Site of disease** Lung Liver Lymph node Bone	no no no yes	no no yes yes	no no yes yes	no yes yes yes	no no yes no	no no no yes	no no yes yes	no no yes yes	no no yes yes	no no yes yes
**PSA level at time of switch (ng/mL)**	520	40	66	20	460	7.3	150	12	1700	210
**Median time since diagnosis (years)**	3	3	26	3	8	5	1	4	6	15
**Gleason Score at diagnosis**	not known	9	not known	9	9	9	9	not known	9	7a
**Treatment prior to RLT** Prostatectomy Androgen receptor pathway inhibitor Taxane therapy External beam radiation of bone metastases	No 2 (bicalutamide, enzalutamide) Docetaxel, Cabazitaxel No	Yes 1 (enzalutamide) None Yes (1 cycle)	Yes 1 (abiraterone) Docetaxel, Cabazitaxel Yes (1 cycle)	Yes 3 (abiraterone, enzalutamide, apalutamide) None Yes (3 cycles)	Yes 3 (bicalutamide, abiraterone, enzalutamide) Docetaxel Yes (2 cycles)	No 2 (bicalutamide, enzalutamide) None Yes (2 cycles)	No 1 (apalutamide) None No	No 2 (abiraterone, enzalutamide) Docetaxel Yes (1 cycle)	No 1 (enzalutamide) Docetaxel, Cabazitaxel No	Yes 3 (abiraterone, enzalutamide, apalutamide) None Yes (2 cycles)
^**177**^ **Lu-PSMA-I&T** Number of cycles Cumulative activity, GBq Response to I&T PSA prior to I&T, ng/mL PSA nadir during I&T, ng/mL Progress after cycle	2 14.8 cont. progress 380 None cont. progress	6 44.6 init. stable 8.3 8.1 2	6 44.8 init. response 730 23 5	6 44.5 cont. progress 2.0 None cont. progress	6 44.4 init. response 320 45 5	2 14.8 cont. progress 3.2 none cont. progress	4 29.8 init. response 360 10 3	3 22.2 init. response 15 7 4	2 14.8 cont. progress 890 None cont. progress	6 44.8 init. response 28.0 19.0 5

ECOG, Eastern Cooperative Oncology Group; PSA, prostate-specific antigen; RLT, radioligand therapy

### Prior therapy

**Fig. 1:**
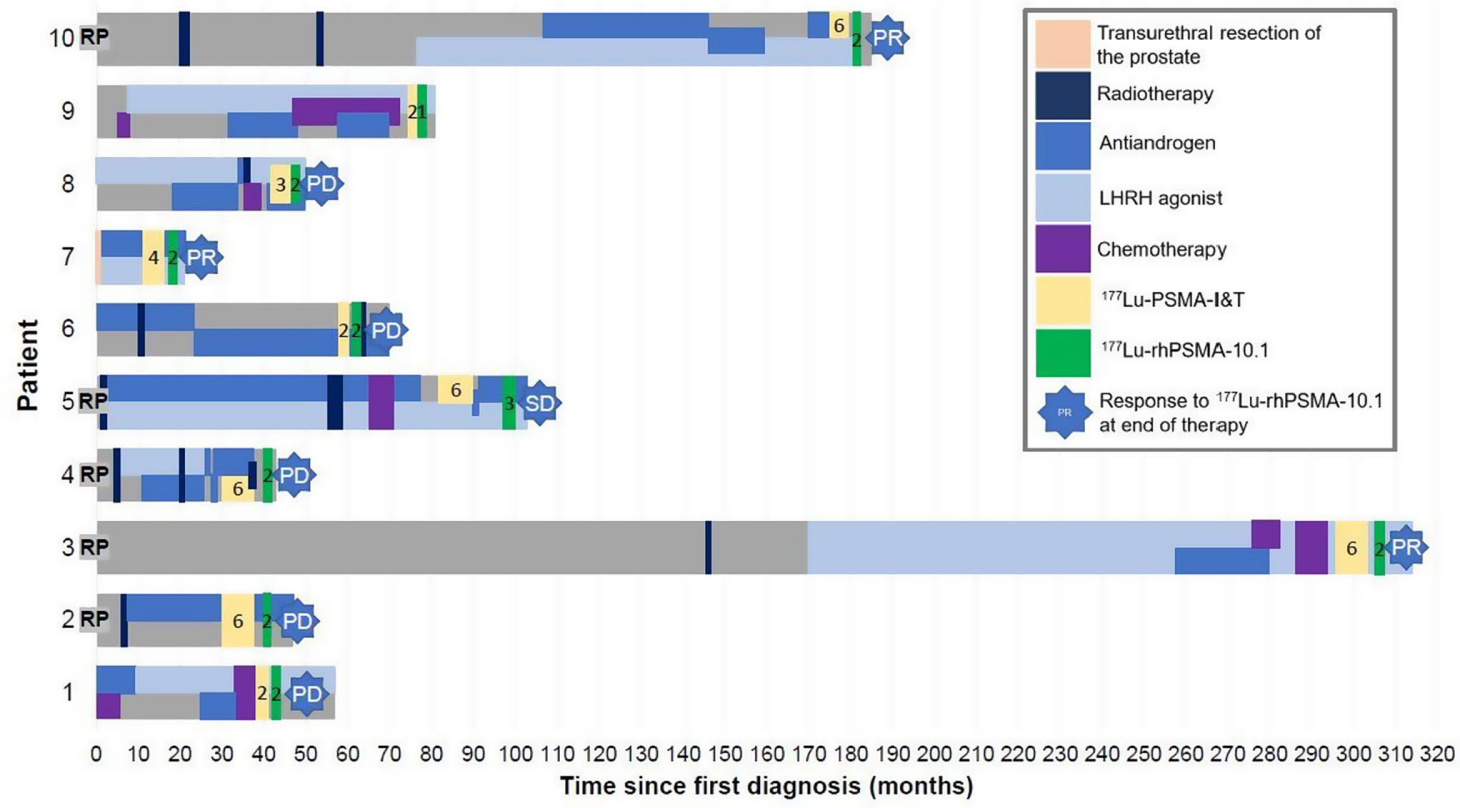
Patients’ treatment before [^177^Lu]Lu-rhPSMA-10.1. Nine of the 10 patients died during follow up, with one patient (Patient 10) remained alive at the time of writing. Number of [^177^Lu]Lu-PSMA-I&T cycles is indicated by number in yellow bar and number of [^177^Lu]Lu-rhPSMA-10.1 cycles is indicated by number in green bar. PD = progressive disease; PR = partial response; SD = stable disease; RP = radical prostatectomy. Radiotherapy was always palliative treatment of bone metastases for pain reduction

Five of the ten patients had previously undergone prostatectomy. None of the patients had primary external beam radiation treatment of the prostate, several patients had EBRT of bone metastases due to pain palliation. The patients’ treatment prior to and including [^177^Lu]Lu-rhPSMA-10.1 is summarized in Table 1; Fig. 1 in detail. Despite the majority of patients initially showing partial response (5/10) or stable disease (1/10) while receiving [^177^Lu]Lu-PSMA-I&T, all patients were ultimately switched to [^177^Lu]Lu-rhPSMA-10.1 after between 2 and 5 cycles of [^177^Lu]Lu-PSMA-I&T (cumulative activity, 14.8–44.8 GBq) due to disease progression under [^177^Lu]Lu-PSMA-I&T.

### [177Lu]Lu-rhPSMA-10.1 rescue therapy

**Fig. 2:**
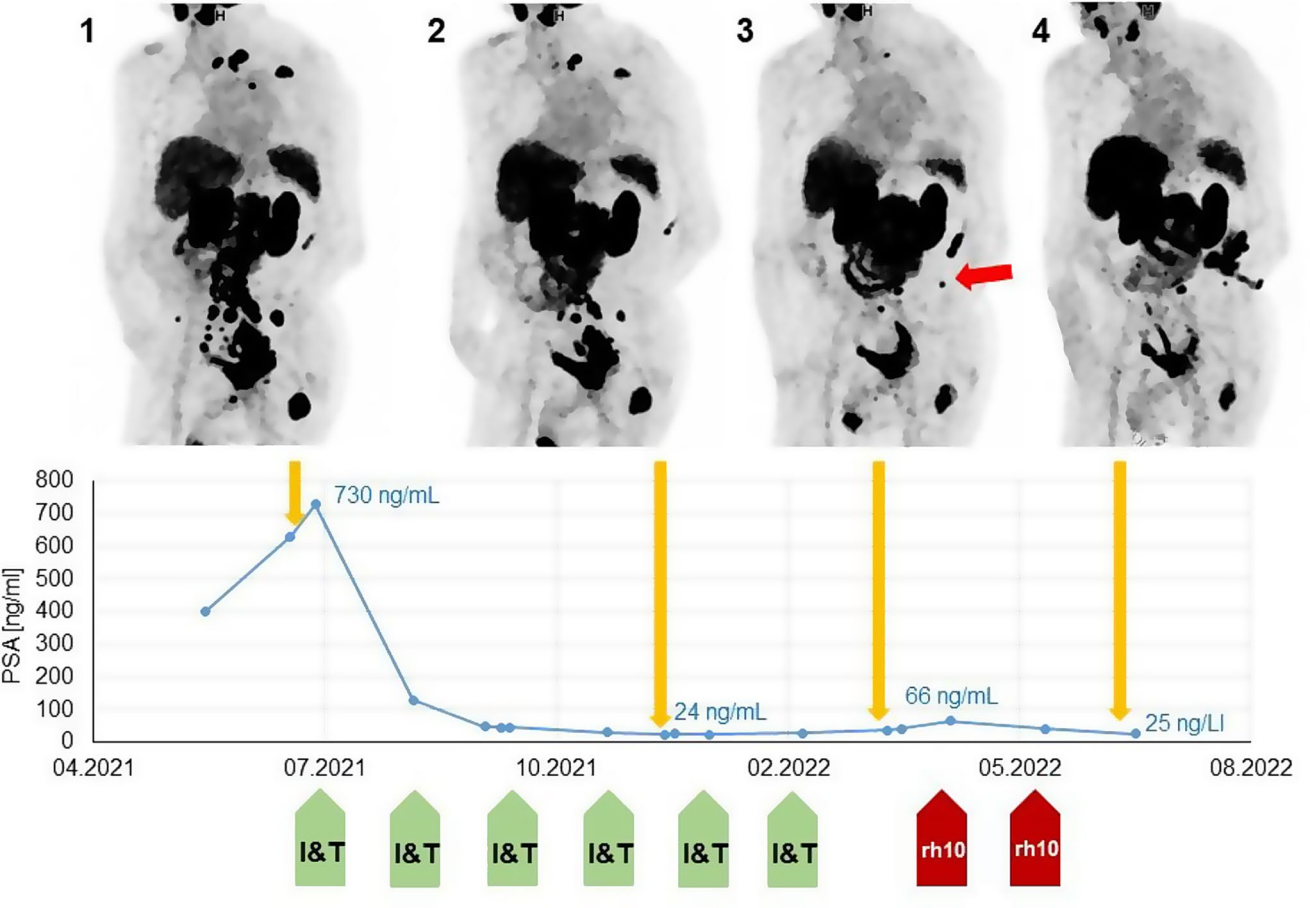
Patient images. The patient (Patient 3 in Table and Fig. 1) initially responding well to treatment with [^177^Lu]Lu-PSMA-I&T with a decline of PSA from 730 ng/mL to 23 ng/mL (PET 1 and 2). A subsequent progression according to PCWG3 was noted, with an increase of PSA level up to 66 ng/mL and new metastases in PET 3 (red arrow), although the overall tumour volume declined between PET 2 and 3. The patient responded well to two cycles of [^177^Lu]Lu-rhPSMA-10.1, with another decrease to PSA 25 ng/mL and a significant reduction of tumour volume on PET

The patients received 1–3 cycles of [^177^Lu]Lu-rhPSMA-10.1 (Table 2). After the first cycle of [^177^Lu]Lu-rhPSMA-10.1, five of the ten patients (50%) showed a response, with a decrease in PSA levels of 49.2% in mean with a standard deviation of 24.1% (range 20–84%). Figure 2 provides a case example from one of these patients. Of the five patients showing a PSA response after their first [^177^Lu]Lu-rhPSMA-10.1 cycle, four had initially responded to ^177^Lu-PSMA-I&T but had subsequently progressed during later cycles. The fifth patient had shown a continuous PSA progression while undergoing treatment with [^177^Lu]Lu-PSMA-I&T from 890 ng/mL to 1700 ng/mL, but rescue therapy with [^177^Lu]Lu-rhPSMA-10.1 provided an immediate PSA response, with levels decreasing to 1300 ng/mL after the first cycle.

**Table 2:** [^177^Lu]Lu-rhPSMA-10.1 rescue therapy and response

Patient	1	2	3	4	5	6	7	8	9	10
[^177^Lu]Lu-rhPSMA-10.1 Number of cycles Cumulative activity, GBq	2 15	2 14.8	2 14.9	2 14.8	3 22.2	2 15.1	2 14.8	2 14.8	1 7.4	2 15.7
**Greatest PSA decrease in response to [**^**177**^**Lu]Lu-rhPSMA-10.1**,** %**	–	–	62	20	–	–	56	–	24	84
**Best response to [** ^**177**^ **Lu]Lu-rhPSMA-10.1 (RECIP)**	Progressive Disease	Progressive Disease	Partial response	Progressive Disease	Stable Disease	Progressive Disease	Partial response	Progressive Disease	n.a.*	Partial response
**Response to [** ^**177**^ **Lu]Lu-rhPSMA-10.1 at the end of therapy (RECIP)**	Progressive Disease	Progressive Disease	Partial response	Progressive Disease	Stable Disease	Progressive Disease	Partial response	Progressive Disease	n.a.*	Partial response
**Overall survival after commencing [**^**177**^**Lu]Lu-rhPSMA-10.1**,** months**	14	7	9	3	6	9	3	3	3	4

PSA, prostate-specific antigen; RECIP, response evaluation criteria in PSMA PET/CT, *not applicable as patient died (not tumour specific) before first imaging

Radiographic response according to RECIP criteria was assessed following the second cycle of [^177^Lu]Lu-rhPSMA-10.1 rescue therapy. For one of the ten patients (10%), no radiographic assessment of response was possible as he died due to an ischemic stroke shortly before undergoing a second treatment cycle. Three of the 10 patients (30%) showed a radiographic partial response. Of these 3 patients, one is still alive at the time of writing (follow-up time 4 month). The other two showed an overall survival of 9 months and 3 months after commencing [^177^Lu]Lu-rhPSMA-10.1 rescue therapy, with the cause of death determined to be unrelated to tumour progression in either case. Altogether, the median OS in our cohort (*n* = 9 patients died before end of data analysis) was 6 month with a range between 3 month and 14 month. In six cases, the death was tumor-related.

### Safety

[^177^Lu]Lu-rhPSMA-10.1 was generally well tolerated (Table 3) among these heavily pre-treated patients with mCRPC. One patient (10%) showed grade 3 leukopenia and grade 4 thrombocytopenia; however, the patient was known to be experiencing grade 2 thrombocytopenia prior to initiation of [^177^Lu]Lu-rhPSMA-10.1 rescue therapy. Another two patients showed grade 3 anaemia, but both patients had progression of intensive bone marrow metastases which was the most probable cause of the anaemia. None severe toxicity to the kidneys (maximum grad 2) or the salivary glands (maximum grade 1) was observed. As stated before, nine of the ten patients died during the follow-up period either due to tumour progression (*n* = 6) or for unrelated reasons (*n* = 3). One patient (Patient 10) is still alive.

**Table 3:** Frequency of adverse events

Adverse event category (CTCAE v 5.0) n of patients (*N* = 10)	Grade 1	Grade 2	Grade 3	Grade 4
Anaemia	4	3	2*	-
Leukopenia	0	0	1	0
Thrombocytopenia	2	0	0	1**
Salivary gland toxicity	9	0	0	0
Decline in kidney function	0	3	0	0
Hepatotoxicity	0	0	0	0

*Likely due to progression of extensive bone marrow metastasis

**The patient was experiencing Grade 2 thrombocytopenia at baseline

## Discussion

The treatment landscape for mCRPC has undergone radical change in recent years, with the current standard approach comprising a combination of anti-androgen therapy, chemotherapy, secondary hormonal therapies, and immunotherapy [15]. ^177^Lu-labelled radiopharmaceuticals such as the recently FDA-approved [^177^Lu]Lu-PSMA-617 provide a further option if the cancer is PSMA-positive. [^177^Lu]Lu-PSMA-617 has been shown to improve overall survival in patients with progressive mCRPC previously treated with androgen receptor inhibitors and taxane chemotherapy [2], and an alternative ^177^Lu-labelled PSMA ligand, [^177^Lu]Lu-PSMA-I&T, shows comparable favourable efficacy and safety profile in the same setting [16].

However, despite the growing armamentarium of therapeutic options for the management of mCRPC, a need exists for further or improved options for those patients who still show disease progression once all standard approaches have been exhausted.

The next-generation radioligand therapy, [^177^Lu]Lu-rhPSMA-10.1, has been developed with optimized properties for therapeutic use. Wurzer et al. have shown that even small modifications to the structure of rhPSMA radiopharmaceuticals can bring remarkable changes to their biodistribution [3], and their work has led to the selection of [^177^Lu]Lu-rhPSMA-10.1, which makes use of a DOTA metal chelator and has the diaminopropionic acid branching unit in the d-Dap stereoconfiguration, as the lead rhPSMA molecule for therapeutic use [17].

[^177^Lu]Lu-rhPSMA-10.1 has undergone extensive preclinical evaluations which show it to have a favourable therapeutic profile, with a high tumour-to-kidney dose ratio [17, 18]. Preclinical studies highlight a number of factors that likely contribute to this favourable biodistribution, including binding affinity, internalization, lipophilicity, net charge, and the extent to which human serum albumin binding occurs. However, intriguingly, there are several examples in the literature of “improved” radioligand therapy candidates in animal models, which fail to translate this potential to the human patient setting. This suggests that the in vitro properties of the radioligand therapy are perhaps less significant than the human specific pharmacokinetics which, of course, cannot be replicated in mice. Reassuringly, the first clinical data with [^177^Lu]Lu-rhPSMA-10.1 from an intra-patient comparison with [^177^Lu]Lu-PSMA-I&T in patients with mCRPC show [^177^Lu]Lu-rhPSMA-10.1 to have a more favourable tumour-to-kidney therapeutic index than [^177^Lu]Lu-PSMA-I&T and is supportive of the preclinical observations [6]. The way this improved profile is achieved seems to relate to achieving a very long tumour effective half-life, without a proportional increase in retention in the normal organs. However, the exact mechanism underlying the long tumour retention remains unclear and is not purely a function of increased plasma half-life.

Among the same first cohort of patients to receive radioligand therapy with this novel radiopharmaceutical, promising efficacy data were achieved, with, a 35–100% PSA response was observed after 4–6 treatment cycles [10]. In order to explore if [^177^Lu]Lu-rhPSMA-10.1 may be of benefit to patients who were showing disease progression despite exhausting all standard treatment options, including with [^177^Lu]Lu-PSMA-I&T, here we report data on [^177^Lu]Lu-rhPSMA-10.1 rescue therapy from a compassionate use program in Germany.

Importantly, this population of patients developed resistance to [^177^Lu]Lu-PSMA-I&T during dosing and were switched to [^177^Lu]Lu-rhPSMA-10.1 based on this observation. We contrast this with some experiences in the literature whereby patients with good responses to therapy return for further re-challenge therapy several months later and likely represent a different patient population.

While some of the patients in this experience initially showed a partial response (5/10) or stable disease (1/10), before subsequently becoming unresponsive to [^177^Lu]Lu-PSMA-I&T, 4/10 never showed any response to [^177^Lu]Lu-PSMA-I&T. Such a complete lack of response to [^177^Lu]Lu-PSMA-I&T highlights the importance of identifying predictive factors indicative of successful outcomes prior to initiation of ^177^Lu-PSMA-based radioligand therapy, such as through use of nomograms, radiomics and artificial intelligence based on pre-therapeutic imaging [20–22]. Notably, one patient with primary resistance to [^177^Lu]Lu-PSMA-I&T, as illustrated by a continuous PSA progression from 890 to 1700 ng/mL while undergoing [^177^Lu]Lu-PSMA-I&T radioligand therapy, showed an immediate response to [^177^Lu]Lu-rhPSMA-10.1 rescue therapy. His PSA decreased after the first cycle to 1300 ng/mL. Unfortunately, this patient suffered from a fatal ischemic stroke shortly before the second treatment cycle, so no further follow-up, including imaging to confirm radiographic response, was possible. As data with [^177^Lu]Lu-PSMA-617 in mCRPC patients show, the ability to deliver a higher radiation dose to the tumour results in greater efficacy [7, 22], and as our previous data show, [^177^Lu]Lu-rhPSMA-10.1 delivers a significantly higher radiation dose to the tumour compared with [^177^Lu]Lu-PSMA-I&T [6], potentially overcoming primary or acquired radiation resistance and thus enabling a response to [^177^Lu]Lu-rhPSMA-10.1 after progression on [^177^Lu]Lu-PSMA-I&T. Together, this perhaps suggests that not all PSMA-based radioligand therapy agents are equal and that failure to respond to one agent should not preclude rechallenge or rescue therapy with another. However, data from a multicenter retrospective analysis of patients who received [^177^Lu]Lu-PSMA-617 or [^177^Lu]Lu-PSMA-I&T, either as extended continuous treatment (*n* = 43), or as a rechallenge (*n* = 68), show that a treatment break preserved the efficacy of ^177^Lu-labelled radioligand therapy [23], Moreover, rates of those showing a 50% PSA decline were significantly higher in the rechallenge group than in the continuous group (57/63 [90%] versus 26/42 [62%]; *P* = 0.006). Although we note the median therapy–free interval was longer (7.2 months) than in the present study and likely represents a different patient population with more favourable disease, it remains possible that the short break in [^177^Lu]-PSMA-I&T therapy (6–12 weeks) may also have prompted a renewed response among our cohort. This should be evaluated further in future studies.

^177^Lu-labelled radiopharmaceuticals are generally well tolerated [6, 9, 10], however, the kidneys remain one of the most important normal organs to consider when planning radioligand therapy due to the risk of delayed radiation nephropathy [24]. Although appropriate renal radiation dose limits are yet to be established for patients undergoing radioligand therapy with newly established beta emitting radiopharmaceuticals, minimizing the radiation exposure to the kidney while maximizing the effective tumour dose should be a key consideration when selecting an agent for radioligand therapy [6]. In this cohort the conventionally defined kidney dose limits (23 Gy) were likely exceeded in several patients. While we did observe three cases (30%) of grade 2 nephrotoxicity without any more severe decline of kidney function in our cohort, we found severe bone marrow toxicity in one case of grade 4 thrombocytopenia (10%), one case of grade 3 leukopenia (10%) and two cases of grade 3 anaemia (20%). This is more than found before in primary RLT using [^177^Lu]-PSMA-I&T for which in a study including 100 patients grade 3/4 toxicities were 4% for thrombocytopenia, 6% for neutropenia and 9% for anaemia [1]. Similar magnitudes have been reported for [^177^Lu]-PSMA-617 [2]. However, despite the low number of patients, the patients in our cohort were heavily pre-treated. The patient suffering from grade 4 thrombocytopenia was already presenting with grade 2 thrombocytopenia prior to [^177^Lu]Lu-rhPSMA-10.1 therapy. In general, this is a common finding in such cohorts, as patients have already undergone treatment with a large number of potentially bone marrow toxic treatments prior to [^177^Lu]Lu-rhPSMA-10.1, including taxane-based chemotherapy and [^177^Lu]Lu-PSMA-I&T. Therefore, it should be expected that a higher level of toxicity might be observed [10].

There are some limitations to the present work. Data are reported from only a small case series of patients who were the first to receive rescue therapy with [^177^Lu]Lu-rhPSMA-10.1 as part of a compassionate use program at our clinic. While the findings suggest that rechallenge with another radioligand may be beneficial despite previous progression on another agent, this should be confirmed in future prospective clinical trials.

## Conclusion

Our preliminary clinical data show that patients with primary or acquired resistance to [^177^Lu]Lu-PSMA-I&T radioligand therapy, as demonstrated by tumour progression, may benefit from a change in treatment to the novel radiohybrid PSMA ligand, [^177^Lu]Lu-rhPSMA-10.1. The higher tumour absorbed radiation doses delivered with [^177^Lu]Lu-rhPSMA-10.1 may help overcome such radiation resistance. Further evaluation of this concept is warranted in larger patient cohorts and prospective settings.

## Data Availability

The datasets generated during and/or analysed during the current study are available from the corresponding author on reasonable request.
